# Sex Differences in Tolerance to Delta-9-Tetrahydrocannabinol in Mice With Cisplatin-Evoked Chronic Neuropathic Pain

**DOI:** 10.3389/fmolb.2021.684115

**Published:** 2021-06-25

**Authors:** Angela N. Henderson-Redmond, LaTaijah C. Crawford, Diana E. Sepulveda, David E. Hale, Julia J. Lesperance, Daniel J. Morgan

**Affiliations:** ^1^ Department of Biomedical Sciences, Marshall University, Huntington, WV, United States; ^2^ Department of Pharmacology, Penn State University College of Medicine, Hershey, PA, United States; ^3^ Department of Anesthesiology and Perioperative Medicine, Penn State University College of Medicine, Hershey, PA, United States; ^4^ Department of Neural and Behavioral Sciences, Penn State University College of Medicine, Hershey, PA, United States

**Keywords:** sex-differences, chronic pain, tolerance, cisplatin, mice, tetrahydrocannabinol, cannabinoids

## Abstract

Tolerance to the pain-relieving effects of cannabinoids limits the therapeutic potential of these drugs in patients with chronic pain. Recent preclinical research with rodents and clinical studies in humans has suggested important differences between males and females in the development of tolerance to cannabinoids. Our previous work found that male mice expressing a desensitization resistant form (S426A/S430A) of the type 1 cannabinoid receptor (CB_1_R) show delayed tolerance and increased sensitivity to the antinociceptive effects of delta-9-tetrahydrocannabinol (∆^9^-THC). Sex differences in tolerance have been reported in rodent models with females acquiring tolerance to ∆^9^-THC faster than males. However, it remains unknown whether the S426A/S430A mutation alters analgesic tolerance to ∆^9^-THC in mice with chemotherapy-evoked chronic neuropathic pain, and also whether this tolerance might be different between males and females. Male and female S426A/S430A mutant and wild-type littermates were made neuropathic using four once-weekly injections of 5 mg/kg cisplatin and subsequently assessed for tolerance to the anti-allodynic effects of 6 and/or 10 mg/kg ∆^9^-THC. Females acquired tolerance to the anti-allodynic effects of both 6 and 10 mg/kg ∆^9^-THC faster than males. In contrast, the S426A/S430A mutation did not alter tolerance to ∆^9^-THC in either male or female mice. The anti-allodynic effects of ∆^9^-THC were blocked following pretreatment with the CB_1_R antagonist, rimonabant, and partially blocked following pretreatment with the CB_2_R inverse agonist, SR144528. Our results show that disruption of the GRK/β-arrestin-2 pathway of desensitization did not affect sensitivity and/or tolerance to ∆^9^-THC in a chronic pain model of neuropathy.

## Introduction

Chemotherapy-evoked neuropathic pain (CENP) is a dose-limiting adverse effect occurring in up to 90% of individuals receiving neurotoxic chemotherapy (Fallon and Colvin, 2013), including the platinum-based cisplatin (Roelofs et al., 1984; Van Der Hoop et al., 1990). As cisplatin does not cross the blood-brain barrier, cisplatin primarily damages peripheral tissues-including dorsal root ganglia and sensory fibers (Gregg et al., 1992), resulting in the development of peripheral neuropathy. Common symptoms of sensory neuropathy include numbness, tingling, and burning pain typically originating in the feet. While sensory neuropathies may spontaneously resolve over time in some patients, in others, it becomes chronic. While treatment options for CENP range from anticonvulsants, antidepressants, and topical treatment, including lidocaine and capsaicin, opioids remain the treatment gold standard (for a review, see Fallon and Colvin, 2013). However, opioids display limited efficacy in the context of neuropathic pain (Arnér and Meyerson, 1988; Dellemijn, 1999) while retaining significant abuse liability.

Cannabinoids represent a viable alternative to opioids for chronic, neuropathic pain management. The endocannabinoid system plays an important role in pain modulation (for a review, see Walker et al., 2001) and cannabinoid (CB) drugs can induce analgesia through activation of two CB receptors, the cannabinoid type-1 (CB_1_; Matsuda et al., 1990) and the cannabinoid type-2 (CB_2_; Munro et al., 1993) receptors. CB_1_ receptors are expressed throughout the central nervous system while CB_2_ receptors are expressed mostly in immune cells (Pertwee, 1997). Rodent models of cisplatin-evoked neuropathy demonstrate cisplatin-mediated increases in mechanical hyperalgesia and allodynia (Joseph and Levine, 2003) and decreased peripheral nerve conduction (Authier et al., 2003). Systemic administration of endocannabinoids [anandamide; (Khasabova et al., 2012)], select CB_1_ [ACEA (Vera et al., 2013)] and CB_2_ [JWH133 (Vera et al., 2013); AM1710 (Deng et al., 2012)]; and mixed CB_1_/CB_2_ agonists [WIN55,212-2 (Vera et al., 2013; Nealon et al., 2019) and CP55,940 (Henderson-Redmond et al., 2020)] have all been shown to attenuate cisplatin-induced mechanical allodynia preclinically. Interestingly, while both cannabidiol (CBD) and Δ^9^-tetrahydrocannabinol (Δ^9^-THC), were able to attenuate mechanical allodynia in paclitaxel-treated mice, CBD (but not Δ^9^-THC) was able to attenuate mechanical allodynia in oxaliplatin-treated mice while Δ^9^-THC (but not CBD) attenuated mechanical allodynia in vincristine-treated mice (King et al., 2017). Taken together, these findings suggest a role for cannabinoids in mediating CENP.

Tolerance to Δ^9^-THC, a mixed CB_1_/CB_2_ agonist, has been demonstrated in humans (Jones et al., 1981; Haney et al., 1999; D’Souza et al., 2008; Gorelick et al., 2012; Cuttler et al., 2016) and pre-clinical rodent models (Bass and Martin, 2000; Morgan et al., 2014; Wakley et al., 2014b; Nealon et al., 2019; Henderson-Redmond et al., 2020; Wiley et al., 2021). Previous work has shown that desensitization of the CB_1_ receptor represents one potential neuroadaptation that can mediate tolerance to cannabinoids (Jin et al., 1999; Martin et al., 2004; Selley et al., 2004; Daigle et al., 2008; Nguyen et al., 2012). CB_1_ receptor-mediated desensitization occurs via phosphorylation by a G protein-coupled receptor kinase (GRK) and consequent recruitment of β-arrestin-2 (Sim et al., 1996; Nguyen et al., 2012). Ensuing studies determined that mutating two serine residues (S426 and S430) to alanines in the carboxy terminus of the CB_1_ receptor tail prevented CB_1_ receptor desensitization *in vitro* (Jin et al., 1999; Daigle et al., 2008). Subsequent *in vivo* studies revealed that male mice expressing the S426A/S430A mutations were more sensitive to and displayed slower tolerance to the antinociceptive effects of Δ^9^-THC in acute models of thermal (tail-flick; Morgan et al., 2014; Henderson-Redmond et al., 2020) and inflammatory (formalin; LaFleur et al., 2018) pain. However, it remains unknown whether these mutations, which disrupt GRK/β-arrestin-2 desensitization of the CB_1_ receptor, likewise mediate tolerance to Δ^9^-THC in a chemotherapy-evoked model of chronic, neuropathic pain.

Women have a higher prevalence of developing neuropathic pain compared to men (Rosen et al., 2017; Fillingim et al., 2009; Mogil, 2012). In addition, more females than males report using Δ^9^-THC for medical purposes (Cuttler et al., 2016). Although a greater number of men present with cannabinoid use disorders (CUDs; Hernandez-Avila et al., 2004; Khan et al., 2013), women tend to show a more rapid progression from first use to dependence, an effect termed “telescoping” (Hernandez-Avila et al., 2004; Ehlers et al., 2010). Sex has been shown to modulate the rate of tolerance to the acute antinociceptive effects of Δ^9^-THC in the tail-flick assay, with female rodents displaying more rapid tolerance than their male littermates (Wakley et al., 2014b). Therefore, it is imperative to identify sex differences that influence both efficacy and tolerance for the effects of Δ^9^-THC in chronic, neuropathic pain. Previously, we have shown that disruption of GRK/β-arrestin-2-induced desensitization of CB_1_ receptors using S426A/S430A mutant mice delays tolerance to the effects of Δ^9^-THC in acute and inflammatory pain. The goal of the current study was to determine whether tolerance was also delayed for the effects of Δ^9^-THC in these mutant mice for cisplatin-evoked neuropathic pain, and whether the mechanisms of cannabinoid tolerance might be sex-specific.

## Materials and Methods

### Subjects

Subjects included 159 experimentally naïve age-matched (10–16 weeks; 20–35 g) adult male (*N* = 72) and female (*N* = 87) S426A/S430A mutant (KI; *N* = 27) and wild-type (WT; *N* = 132) mice backcrossed for 10^+^ generations onto a C57BL/6J background. Desensitization-resistant S426A/S430A mice were created as previously described by replacing serines 426 and 430 with alanines in the carboxy terminal of the CB_1_ receptor (Morgan et al., 2014). Mice were group housed (3–5/cage) during all studies and kept on a 12:12 h light/dark cycle (lights out at 18:00) with *ad libitum* access to food and water. Female mice, while group housed, were not monitored for estrus cycle. Mice were weighed daily prior to drug administration to ensure proper dosing. Animal care procedures were conducted in accordance with NIH guidelines for the Humane Care and Use of Laboratory Animals (2015) and with approval from the Pennsylvania State University and Marshall University Institutional Animal Care and Use Committees (IACUC).

### Drugs/Materials

Delta-9-tetrahydrocannabinol (∆^9^-THC) was obtained from the National Institute on Drug Abuse Drug Supply Program (Bethesda, MD, United States). The selective CB_1_ receptor inverse agonist rimonabant (SR141716; Rinaldi-Carmona et al., 1994), and the CB_2_ receptor-selective inverse agonist, SR144528 (Rinaldi-Carmona et al., 1998), were obtained from the Cayman Chemical Company (Ann Arbor, MI, United States). For all experiments, ∆^9^-THC, rimonabant, and SR144528 were dissolved in 0.9% saline, 5% Cremaphor EL, and 5% ethanol (18:1:1 v/v/v) and administered intraperitoneally (IP) using an injection volume of 10 ml/kg. Drug injections were given either 30 or 60 min (see below) prior to testing. Cisplatin was obtained from Tocris (Minneapolis, MN, United States), dissolved in 0.9% physiological saline, and administered IP immediately following subcutaneous (SC) administration of 1 ml of 4% sodium bicarbonate (Fisher Scientific, Pittsburgh, PA, United States) solution dissolved in 0.9% saline. The selection of Δ^9^-THC, rimonabant, and SR144528 doses were based on prior work in our lab (Yuill et al., 2017; Henderson-Redmond et al., 2020).

### Cisplatin-Induced Neuropathy and Von Frey Testing

Neuropathic pain was induced in male (*N* = 72) and female (*N* = 87) S426A/S430A and wild-type littermates with four weeks of once-weekly injections of 5 mg/kg cisplatin (IP). To prevent renal damage and lethality from cisplatin due to nephrotoxicity, mice were co-administered 1 ml of a 4% sodium bicarbonate solution (SC) prior to treatment with cisplatin (Guindon et al., 2014). To confirm the establishment of a neuropathic pain state, mechanical allodynia was assessed prior to and after cisplatin treatment using an electronic von Frey anesthesiometer equipped with a semi-flexible polypropylene super-tip (IITC Life Science Inc., Woodland Hills, CA, United States).

Mice were acclimated in small acrylic chambers (2.5ʺ × 4ʺ × 3.5ʺ) on a wire mesh table for 20 min prior to von Frey testing. Mechanical allodynia was assessed by measuring the amount of force (in grams) applied to the right hind paw that was required to elicit a paw withdrawal response. Measurements were made in triplicate with an interval of ∼3–5 min between testing trials and the average value was calculated. Pre- and post-baseline measurements of mechanical allodynia were assessed prior to and after cisplatin treatment. Mice that did not exhibit at least a 40% reduction in pre-vs. post-baseline measurements of mechanical allodynia after the last cisplatin treatment were considered non-neuropathic and excluded from the study (∼4% of mice). Neuropathic mice were injected (IP) once-daily with either vehicle (18:1:1), 6 mg/kg, or 10 mg/kg ∆^9^-THC 60 min prior to von Frey testing. For dose response testing, female wild-type mice were injected once-daily and assessed for mechanical allodynia 60 min following treatment with vehicle (0; 18:1:1; day 1), 0.3 mg/kg (day 1), 1 mg/kg (day 2), 3 mg/kg (day 3), 10 mg/kg (day 4), and 30 mg/kg (day 5) of ∆^9^-THC to determine the ∆^9^-THC dose that could fully reverse cisplatin-induced allodynia.

### Use of CB_1_ and CB_2_ Receptor Antagonists

Since ∆^9^-THC is a mixed cannabinoid agonist at both CB_1_ and CB_2_ receptors, the goal of this experiment was to determine the extent to which the anti-allodynic effects of ∆^9^-THC were mediated by each of those receptors. This was accomplished with a separate group of neuropathic male (*N* = 16) and female (*N* = 16) wild-type mice, assessed using a within-subjects design, to determine the effects of Vehicle (18:1:1; Veh), 10 mg/kg rimonabant (SR141716; CB_1_A inverse agonist), and 10 mg/kg SR144528 (CB_2_ inverse agonist; CB_2_A) alone or in combination with 10 mg/kg ∆^9^-THC. The dose of 10 mg/kg ∆^9^-THC was chosen because it fully reversed mechanical allodynia in both male and female mice. Mice were treated once-weekly (Wednesdays) with one of the following six IP injection combinations (Veh/Veh; CB_1_A/Veh; CB_2_A/Veh; Veh/∆^9^-THC; CB_1_A/∆^9^-THC; or CB_2_A/∆^9^-THC. All mice were randomly assigned to treatment order. On testing days, mice were first treated (IP) with either: Vehicle, 10 mg/kg CB_1_A, or 10 mg/kg CB_2_A and assessed for mechanical allodynia 30 min later. Immediately after this assessment, mice were then injected (IP) with either vehicle or 10 mg/kg ∆^9^-THC and reassessed for mechanical allodynia with the von Frey test 60 min later. All results were reported as the amount of force (in grams) required to elicit a paw-withdrawal response.

### Data Analyses

Data were analyzed using SPSS version 25.0 (IBM SPSS Statistics, Armonk, NY, United States) and Prism Graph Pad (7.05; GraphPad, La Jolla, CA, United States). Although female mice were used, vaginal smears were not performed to assess estrus cycle stage. The investigator performing the experiment was blinded to mouse genotypes and drug treatment. Two- and three-way analysis of variance (ANOVAs) analyses were run where appropriate with genotype, day/dose, sex, and/or time point as the main factors. Since we were specifically interested in examining whether there were differences in genotype as a function of sex, we followed up three-way ANOVAs with two-way ANOVAs for tolerance experiments. For all repeated measure analyses, Mauchly’s test of sphericity was calculated to assess equal variance. Where sphericity was violated, the Greenhouse-Geisser correction was used to reduce the probability of making a type I error. When the Greenhouse-Geisser correction was used for reporting degrees of freedom, it has been rounded off to the nearest whole number. Bonferroni post-hoc analyses were performed when significant interaction effects were detected. All data are expressed as the mean ± the standard error of the mean (SEM). For all analyses, significance was set at *p* < 0.05.

## Results

### Tolerance to 6 mg/kg Delta-9-Tetrahydrocannabinol in Male and Female S426A/S430A and Wild-Type Mice

**FIGURE 1:**
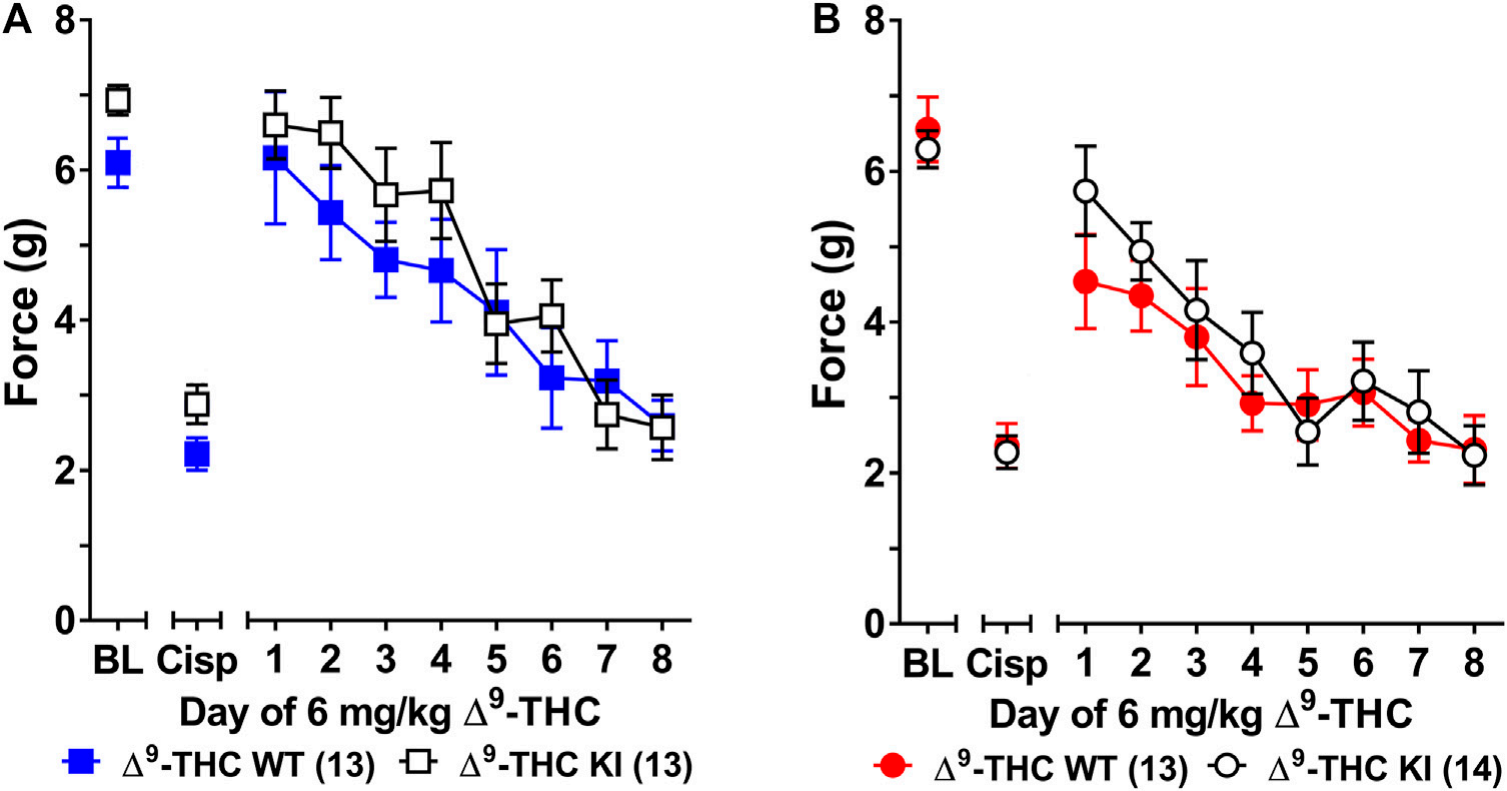
Development of tolerance to the anti-allodynic effects of 6 mg/kg of ∆^9^-THC in male and female wild-type and S426A/S430A mice. Tolerance to the anti-allodynic effects of 6 mg/kg ∆^9^-THC was determined in both male **(A)** and female **(B)** S426A/S430A (KI; unfilled) and wild-type (WT; filled) mice. Mice were assessed via the von Frey assay for the amount of force (in grams) required to elicit a paw withdrawal response 60 min following treatment with 6 mg/kg ∆^9^-THC. BL represents the pre-cisplatin baseline and CISP the post-cisplatin baseline. Error bars represent the mean ± SEM. Each mouse was tested in triplicate and those values averaged to determine a single value for each mouse each day of testing. Data were analyzed using separate two-way ANOVAs with Bonferroni post-hoc tests. Sample sizes for each group (shown in parentheses) include 26 male (13 WT; 13 KI) and 27 female (13 WT; 14 KI) mice.

Tolerance to the anti-allodynic effects of once-daily injections of 6 mg/kg ∆^9^-THC was assessed in male and female S426A/S430A and wild-type mice (Figure 1). Three-way ANOVA (genotype x sex x treatment) indicated that there was a main effect of cisplatin treatment (*F*_1,61_ = 861.6, *p* < 0.001) indicating that once-weekly treatment with 5 mg/kg of cisplatin caused mechanical allodynia associated with chronic neuropathic pain. However, there were no sex (*p* = 0.54) or genotype (*p* = 0.25) differences in either pre-cisplatin (6.40 ± 0.15) or post-cisplatin (2.41 ± 0.12) measures of mechanical allodynia.

**FIGURE 2:**
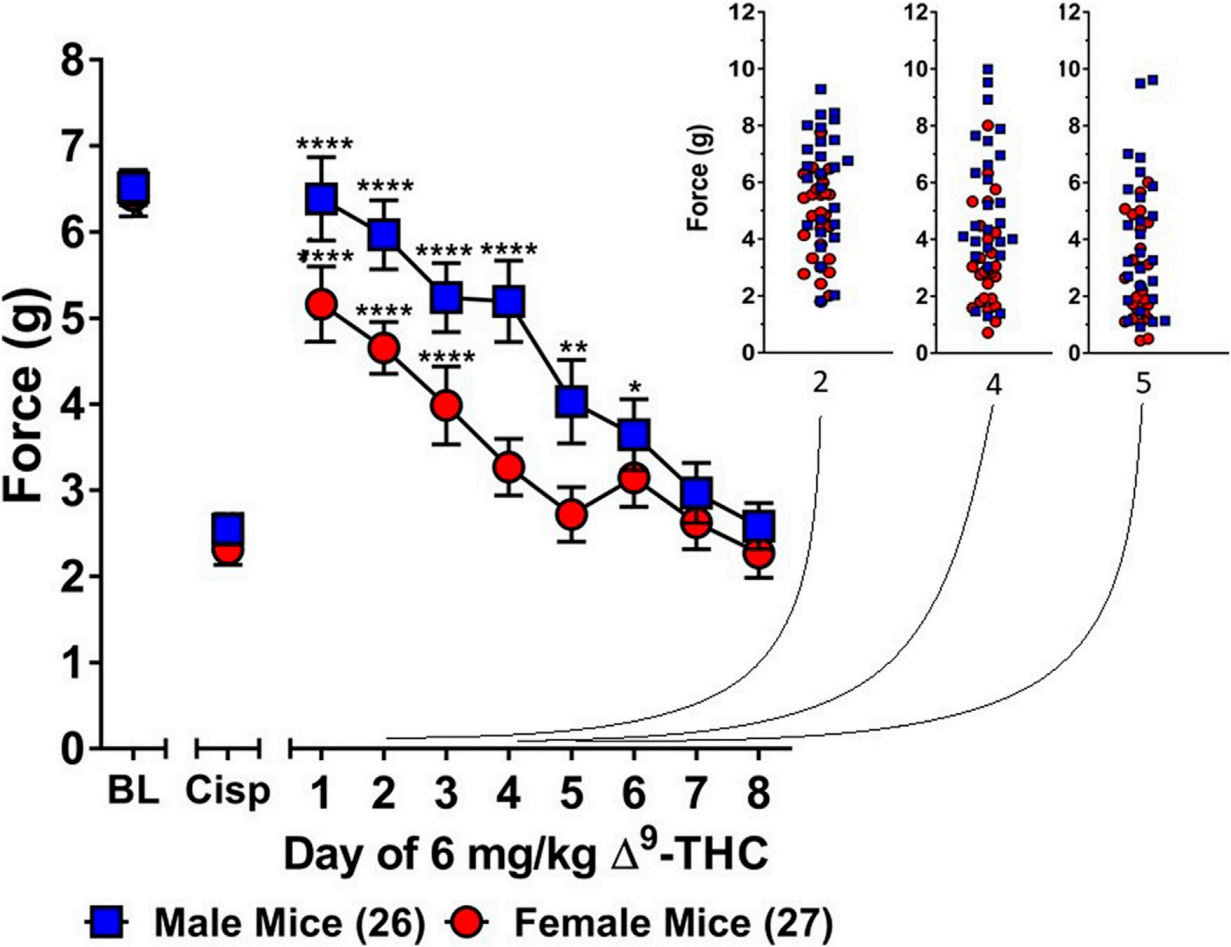
Development of tolerance to the anti-allodynic effects of 6 mg/kg of ∆^9^-THC in female and male mice. Tolerance to the anti-allodynic effects of 6 mg/kg ∆^9^-THC was determined in female (red circles) and male (blue squares) mice collapsed across genotype. Mice were assessed using the von Frey assay for the amount of force (in grams) required to elicit a paw withdrawal response 60 min following treatment with 6 mg/kg ∆^9^-THC. BL represents the pre-cisplatin baseline and CISP the post-cisplatin baseline. Error bars represent the mean ± SEM. Each mouse was tested in triplicate and those values averaged to determine a single value for each mouse each day of testing. Data were analyzed using a two-way ANOVAs with Bonferroni post-hoc tests. [*p < 0.05; **p > 0.01; ***p < 0.001; ****p < 0.0001; compared to the post-cisplatin baseline]. Sample sizes for each group (shown in parentheses) 26 male (13 WT; 13 KI) and 27 female (13 WT; 14 KI) mice.

Three-way ANOVA (sex x genotype x day) was performed to assess tolerance to the anti-allodynic effects of once-daily treatment with 6 mg/kg of ∆^9^-THC in male and female S426A/S430A and wild-type mice with cisplatin-induced neuropathy (Figure 1). Although all mice developed tolerance to the anti-allodynic effects of ∆^9^-THC following 8 days of daily treatment (*F*_7,336_ = 40.91, *p* < 0.001), there was no effect of genotype (*p* = 0.423; Figures 1A,B). There was a main effect of sex (*F*_1,48_ = 5.035, *p* = 0.029) indicating that males (4.44 ± 0.31) showed a greater anti-allodynic response to 6 mg/kg ∆^9^-THC than female littermates (3.47 ± 0.30). There was also a day-by-sex interaction (*F*_7,336_ = 2.250, *p* = 0.030) such that male mice were more sensitive than female mice to the anti-allodynic effects of ∆^9^-THC on days 2 (*p* = 0.014), 4 (*p* = 0.003), and 5 (*p* = 0.043). Comparing males and females to their own baselines, females developed complete tolerance by day 4 and males by day 7 of treatment with 6 mg/kg ∆^9^-THC (Figure 2). Taken together, these finding suggest that female mice were faster to develop tolerance to the anti-allodynic effects of 6 mg/kg ∆^9^-THC than their male counterparts (Figure 2).

Subsequent one-way ANOVAs for male and female wild-type mice indicated that 6 mg/kg ∆^9^-THC caused a complete reversal of neuropathic pain on day 1 in males (6.25 ± 0.24, *p* = 1.00), with tolerance developing following 5 days of treatment. In contrast, mechanical allodynia associated with neuropathic pain was only partially reversed by 6 mg/kg ∆^9^-THC in females on day 1 (5.14 ± 0.43, *p* < 0.05 compared to pre-cisplatin baseline) and they were completely tolerant to the anti-allodynic effects of 6 mg/kg of ∆^9^-THC following 4 days of treatment.

**FIGURE 3:**
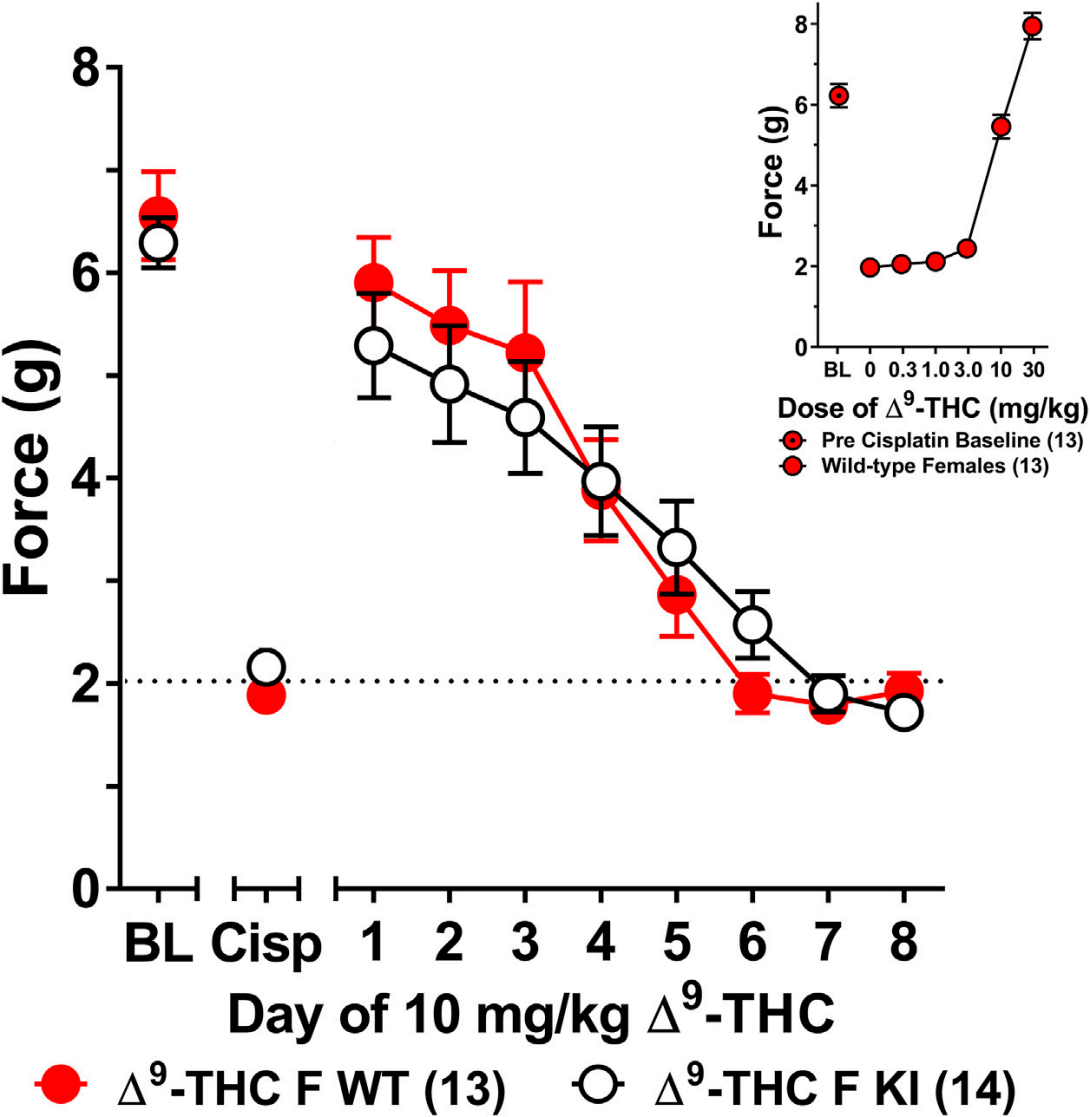
Development of tolerance to the anti-allodynic effects of 10 mg/kg of ∆^9^-THC in female wild-type and S426A/S430A mice. Results from a dose-response (insert) revealed that 10 mg/kg of ∆^9^-THC fully reversed allodynia in wild-type females. Tolerance to the anti-allodynic effects of 10 mg/kg ∆^9^-THC was determined in female S426A/S430A (KI; unfilled) and wild-type (WT; filled) mice. Mice were assessed using the von Frey assay for the amount of force (in grams) required to elicit a paw withdrawal response 60 min following treatment with 6 mg/kg ∆^9^-THC. BL represents the pre-cisplatin baseline and CISP the post-cisplatin baseline. Error bars represent the mean ± SEM. Each mouse was tested in triplicate and those values averaged to determine a single value for each mouse each day of testing. Data were analyzed using separate two-way ANOVAs with Bonferroni post-hoc tests. Sample sizes for each group (shown in parentheses) include 13 female WT mice for the dose response and 13 female WT and 14 female KI mice for daily tolerance.

The choice to use 6 mg/kg of ∆^9^-THC was based on our previous finding that this dose was sufficient to fully reverse cisplatin-evoked mechanical allodynia in male mice (Henderson-Redmond et al., 2020). However, since this dose did not fully reverse mechanical allodynia in female mice, a dose-response curve generated in females (Figure 3 insert) revealed that female mice displayed a complete reversal of cisplatin-induced mechanical allodynia when treated with 10 mg/kg of ∆^9^-THC. As such, female wild-type and S426A/S430A mice were reassessed for differences in tolerance to 10 mg/kg ∆^9^-THC as a function of genotype. Results from a two-way ANOVA assessing tolerance to 10 mg/kg ∆^9^-THC in female S426A/S430A mutant and wild-type mice revealed a main effect of day (*F*_7,175_ = 39.69, *p* < 0.001), but neither a main effect of genotype (*p* = 0.8306) nor a genotype-by-day interaction (*p* = 0.4013). Separate analyses showed that 10 mg/kg ∆^9^-THC resulted in a full reversal of allodynia for both female wild-type and S426A/S430A mice. However, as with 6 mg/kg, there was no difference in the rate of tolerance to 10 mg/kg of ∆^9^-THC as a function of genotype, with both mutants and wild-types showing complete tolerance following 4 days of treatment with 10 mg/kg ∆^9^-THC (Figure 3).

### Tolerance to 10 mg/kg Delta-9-Tetrahydrocannabinol in Male and Female Wild-Type Mice

A second group of male and female wild-type mice were assessed for tolerance to the anti-allodynic effects of 10 mg/kg of ∆^9^-THC, a dose that fully reversed allodynia in both sexes. As there was no difference in tolerance or sensitivity to either 6 and/or 10 mg/kg of ∆^9^-THC in male and female S426A/S430A mice compared to their wild-type counterparts, only wild-type mice were used for all subsequent experiments. In contrast to our previous experiments that did not include a vehicle control, in mice with cisplatin-induced neuropathy, subsequent groups of male and female wild-type mice were injected with vehicle 1 h prior to being assessed by the von Frey test, after which, they were immediately injected with 6 or 10 mg/kg of ∆^9^-THC and assessed again 60 min later to determine the anti-allodynic effects of ∆^9^-THC.

**FIGURE 4:**
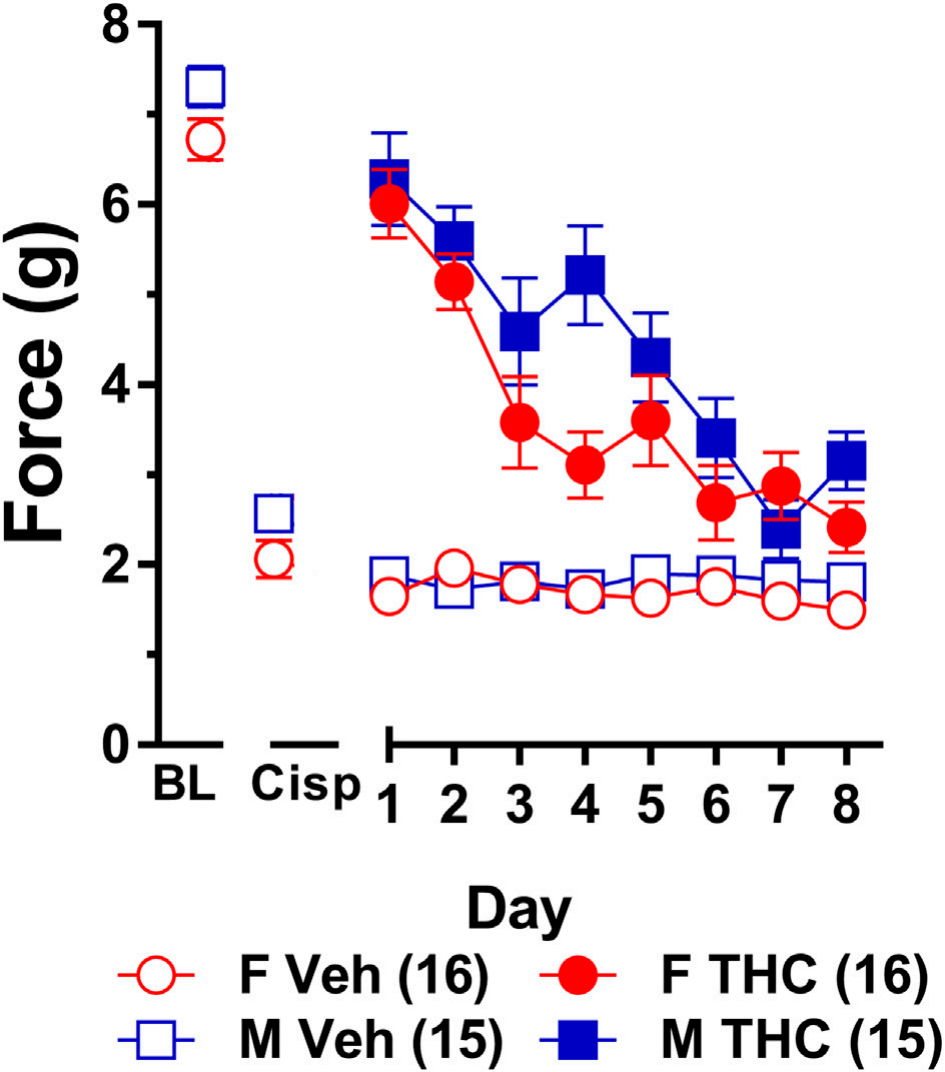
Development of tolerance to the anti-allodynic effects of 10 mg/kg of ∆^9^-THC in male and female wild-type mice. Tolerance to the anti-allodynic effects of vehicle (unfilled) and 10 mg/kg (filled) ∆^9^-THC was determined in both male (squares) and female (circles) wild-type (WT) mice using the von Frey. Mice were first assessed for the amount of force (in grams) required to elicit a paw withdrawal response 60 min following treatment with vehicle and 60 min later following treatment with 10 mg/kg ∆^9^-THC. BL represents the pre-cisplatin baseline and CISP the post-cisplatin baseline. Error bars represent the mean ± SEM. Each mouse was tested in triplicate and those values averaged to determine a single value for each mouse for each dose tested on each day of testing. Data were analyzed using separate two-way ANOVAs with Bonferroni post-hoc tests. Sample sizes for each group (shown in parentheses) include 15 male and 16 female WT mice.

Results from a two-way ANOVA (sex x treatment) showed that there was a main effect of cisplatin treatment (*F*_1,29_ = 88.68, *p* < 0.001) indicating that treatment with cisplatin evoked mechanical allodynia in both sexes (Figure 4). There was also a main effect of sex (*F*_1,29_ = 9.307, *p* = 0.005) indicating that females displayed lower overall von Frey scores than males. There was not a sex-by-treatment interaction effect (*p* = 0.86) indicating that cisplatin-treatment reduced male and female von Frey test responses by approximately the same amount (65% in males; 69% in females). Thus, despite females having slightly lower overall von Frey responses, cisplatin induced approximately the same degree of neuropathy in male and female mice.

Results from a two-way ANOVA (sex x day) examining whether sex altered the rate of tolerance to 10 mg/kg ∆^9^-THC revealed a main effect of day (*F*_7,203_ = 26.49, *p* < 0.001) and a significant sex-by-day interaction (*p* = 0.028). Post-hoc analyses showed that 10 mg/kg ∆^9^-THC was able to fully reverse mechanical allodynia in both males and females and that females showed a decreased anti-allodynic response to this dose of ∆^9^-THC on day 4 compared to males (*p* = 0.005). Compared to day 1 of ∆^9^-THC treatment, females showed evidence of partial tolerance on day 3 whereas males took until day 4 to show evidence of tolerance (Figure 4). Comparison to their own post-cisplatin baseline revealed females were completely tolerant to the anti-allodynic effects of ∆^9^-THC by day 4 of treatment while males did not display full tolerance until day 6 of treatment.

To ensure tolerance to ∆^9^-THC was not an artifact of learned behavior (to withdraw their paw), mice were baselined daily following treatment with vehicle prior to treatment with ∆^9^-THC. Subsequent two-way ANOVAs (treatment x day) were run comparing the daily response of mice following vehicle and ∆^9^-THC across each day. For both males and females, there were main effects of treatment (males: *F*_1,14_ = 80.60, *p* < 0.001; females: *F*_1,15_ = 67.39, *p* < 0.001) and day (males: *F*_7,98_ = 11.17, *p* < 0.001; females: *F*_7,105_ = 15.82, *p* < 0.001), and treatment-by-day interactions (males: *F*_7,98_ = 12.30, *p* < 0.001; females: *F*_7,105_ = 10.86, *p* < 0.001). Post-hoc analyses revealed that these interactions were driven by the difference in anti-allodynic response following treatment with ∆^9^-THC vs. vehicle. Further, male mice returned to their vehicle-treatment baseline after 7 days of 10 mg/kg ∆^9^-THC while females returned to their vehicle-treated baseline after 6 days. These data suggest that when using a dose of ∆^9^-THC (10 mg/kg) capable of completely reversing allodynia in both sexes, females are slightly faster to develop tolerance to the anti-allodynic effects of ∆^9^-THC compared to male littermates (Figure 4).

### Tolerance to Equally Efficacious Doses of Delta-9-Tetrahydrocannabinol in Male and Female Wild-Type Mice

**FIGURE 5:**
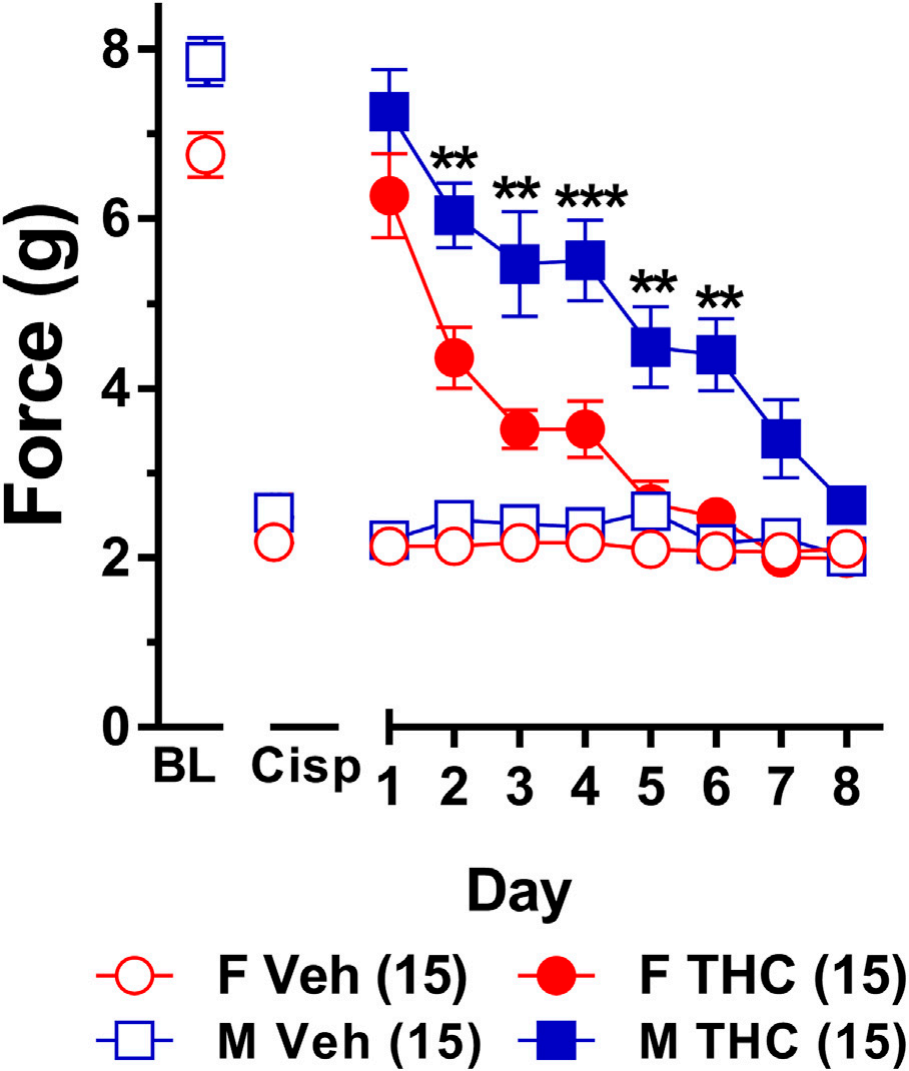
Development of tolerance to equally efficacious doses of ∆^9^-THC in male and female wild-type mice. Tolerance to the anti-allodynic effects of vehicle (unfilled) and equally efficacious doses of ∆^9^-THC (filled) was determined in both male (squares) and female (circles) wild-type (WT) mice using the von Frey. Mice were first assessed for the amount of force (in grams) required to elicit a paw withdrawal response 60 min following treatment with vehicle and 60 min later following treatment with 10 mg/kg (female) or 6 mg/kg (male) of ∆^9^-THC. BL represents the pre-cisplatin baseline and CISP the post-cisplatin baseline. Error bars represent the mean ± SEM. Each mouse was tested in triplicate and those values averaged to determine a single value for each mouse for each dose on each day testing. Data were analyzed using separate two-way ANOVAs with Bonferroni post-hoc tests. (**p > 0.01; ***p < 0.001; compared to females on the same day). Sample sizes for each group (shown in parentheses) include 15 male and 15 female WT mice.

A third group of male and female wild-type mice were assessed for tolerance to the anti-allodynic effects of “equally efficacious doses” of 10 mg/kg ∆^9^-THC in females and 6 mg/kg ∆^9^-THC in males. Results from a two-way ANOVA (sex x treatment) showed that there was a main effect of cisplatin treatment (*F*_1,28_ = 566.5, *p* < 0.001) indicating that treatment with cisplatin evoked mechanical allodynia in both sexes (Figure 5). There was also a main effect of sex (*F*_1,28_ = 10.13, *p* = 0.004) indicating that females displayed lower overall von Frey scores compared to males. However, there was not a sex-by-treatment interaction effect (*p* = 0.08) indicating that cisplatin-treatment reduced male and female von Frey test responses by approximately the same amount (68% in both males and females). Thus, despite having slightly lower overall von Frey responses, cisplatin induced approximately the same degree of neuropathy in our male and female mice.

Results from a two-way ANOVA (sex x day) examining whether sex altered the rate of tolerance to mice given equally efficacious doses of ∆^9^-THC (6 mg/kg in males and 10 mg/kg in females) revealed a main effect of day (*F*_7,196_ = 32.61, *p* < 0.001) and sex (*F*_1,28_ = 33.96, *p* < 0.001) but not a significant sex-by-day interaction (*p* = 0.44). Post-hoc analyses showed that 6 and 10 mg/kg ∆^9^-THC were able to fully reverse mechanical allodynia in male and female mice, respectively. Post-hoc analyses also determined that male and female mice did not differ in von Frey scores at baseline (*p =* 0.30), following treatment with cisplatin (*p* = 0.99), or on day 1 following treatment with equally efficacious doses ∆^9^-THC (*p* = 0.49). Despite the lack of a significant sex-by-day interaction, which suggests that males and females did not differ in the rates at which they developed tolerance to ∆^9^-THC, females showed a rapid and prolonged decrease in response to the anti-allodynic effects of ∆^9^-THC on days 2 (*p* = 0.010), 3 (*p* = 0.001), 4 (*p* = 0.001), 5 (*p* = 0.003), and 6 (*p* = 0.002) compared to their male littermates. Likewise, compared to day 1 of ∆^9^-THC treatment, females showed evidence of partial tolerance on day 2 whereas males took until day 3 to show evidence of tolerance. Finally, comparison to their own post-cisplatin baseline revealed females were completely tolerant to the anti-allodynic effects of ∆^9^-THC by day 5 of treatment while males did not display full tolerance until day 7 of treatment (Figure 5). Taken together, these data suggest that despite a nonsignificant sex-by-day interaction, female mice developed tolerance to the anti-allodynic effects of equally efficacious doses of ∆^9^-THC more rapidly than their male counterparts.

To establish that tolerance to ∆^9^-THC was not an artifact of learned behavior (to withdraw their paw), mice were baselined daily following treatment with vehicle prior to treatment with ∆^9^-THC. Subsequent two-way ANOVAs (treatment x day) were run comparing the daily response of mice following vehicle and ∆^9^-THC across each day. For both males and females, there were main effects of treatment (males: *F*_1,14_ = 152.6, *p* < 0.001; females: *F*_1,14_ = 203.3, *p* < 0.001) and day (males: *F*_7,98_ = 11.44, *p* < 0.001; females: *F*_7,98_ = 22.08, *p* < 0.001), and treatment-by-day interactions (males: *F*_7,98_ = 12.35, *p* < 0.001; females: *F*_7,98_ = 18.72, *p* < 0.001). Post-hoc analyses revealed that these interactions were driven by the difference in anti-allodynic response following treatment with ∆^9^-THC vs. vehicle. Further, male mice returned to their vehicle-treatment baseline after 7 days of 6 mg/kg ∆^9^-THC while females returned to their vehicle-treated baseline after 5 days of 10 mg/kg ∆^9^-THC. These data suggest that when using equally efficacious doses of ∆^9^-THC capable of completely reversing allodynia in both sexes (10 mg/kg in females and 6 mg/kg in males), females are slightly faster to develop tolerance to the anti-allodynic effects of ∆^9^-THC compared to male littermates (Figure 5).

### Use of CB_1_ and CB_2_ Receptor Antagonists

**FIGURE 6:**
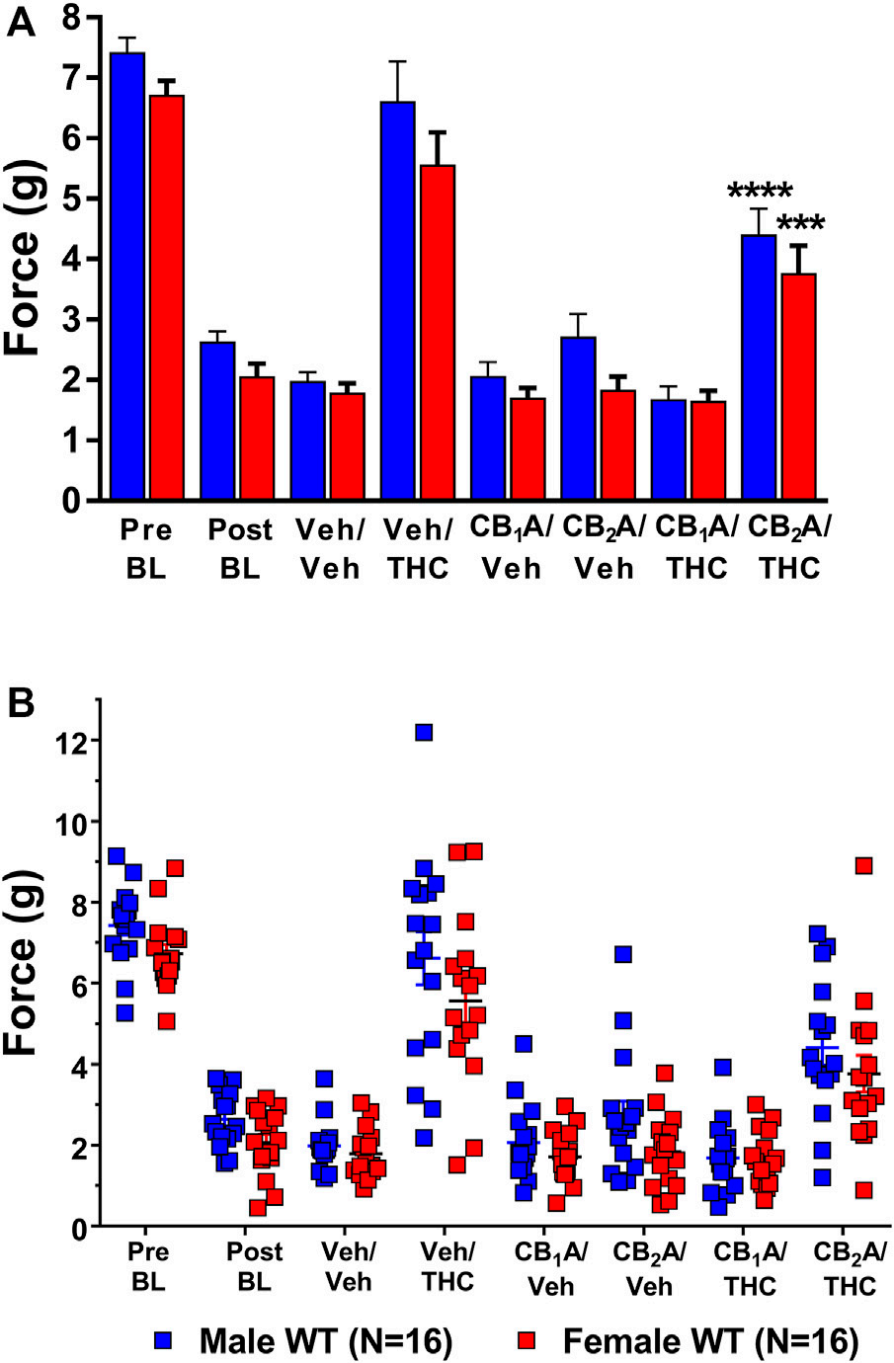
Mediation of the anti-allodynic effects of 10 mg/kg ∆^9^-THC by CB_1_ and/or CB_2_ receptors. **(A)** Aggregate and **(B)** Individual Plots graphs showing male (blue squares) and female (red circle) wild-type (WT) mice showed a full reversal of allodynia following pretreatment with 10 mg/kg of ∆^9^-THC. Mice were treated and then assessed for the amount of force (g) required to elicit a paw withdrawal response 30 min are pretreatment with either vehicle, 10 mg/kg of the CB_1_ receptor inverse agonist SR141716A, or 10 mg/kg of the CB_2_ receptor inverse agonist SR144528 and again 60 min following treatment with either vehicle or 10 mg/kg ∆^9^-THC. Error bars represent the mean ± SEM. Each mouse was tested in triplicate and those values averaged to determine a single value for each mouse each day of testing. Data were analyzed using a two-way ANOVA with Bonferroni post-hoc tests. [***p < 0.001; ****p < 0.0001; compared to both pre- (Pre) and post- (Post) Cisplatin baselines (BL)]. Sample sizes for each dosing combination (shown in parentheses) include 16 male and 16 female WT mice.

Since ∆^9^-THC is a mixed agonist capable of acting at both the CB_1_ and CB_2_ receptors, this experiment determined which receptors mediated the anti-allodynic effects of ∆^9^-THC. Mice were pretreated with either the selective CB_1_ receptor inverse agonist rimonabant, the CB_2_ receptor inverse agonist SR144528, or vehicle prior to treatment with either 10 mg/kg ∆^9^-THC or vehicle. Results from a two-way ANOVA (sex x treatment combination) revealed main effects of sex (*F*_1,30_ = 10.73, *p* = 0.003) and treatment combination (*F*_7,102_ = 86.51, *p* < 0.001) but not a significant interaction (*p* = 0.771; Figure 6).

Post-hoc analysis revealed that treatment with vehicle alone did not differ from the post-cisplatin baseline in either male (*p* = 0.8349) or female (*p* = 0.9988) mice. Treatment with either the CB_1_ receptor inverse agonist rimonabant or the CB_2_ receptor inverse agonist, SR144528, alone also did not differ from vehicle treatment in either male [CB_1_ (*p =* 0.9999); CB_2_ (*p* = 0.7384)] or female [CB_1_ (*p =* 0.9999); CB_2_ (*p* = 0.9999)] mice. Treatment with 10 mg/kg of ∆^9^-THC alone was able to fully reverse mechanical allodynia in both male and female mice to their pre-cisplatin baseline levels, and this effect was completely blocked in both male and female mice by pretreatment with rimonabant. Interestingly, pretreatment with the CB_2_ receptor inverse agonist partially blocked the ability of ∆^9^-THC to reverse these effects (Figure 6). These results suggest a strong role for CB_1_ receptors and a partial role for CB_2_ receptors in mediating the anti-allodynic effects of ∆^9^-THC in mice with cisplatin-induced neuropathic pain. The lack of an interaction effect suggests that sex differences observed in ∆^9^-THC tolerance were not due to differences in cannabinoid receptor mediation.

## Discussion

The first goal of this study was to determine whether blocking the GRK/β-arrestin-2 pathway of desensitization using the S426A/S430A mutant mice altered sensitivity and/or tolerance to ∆^9^-THC in a clinically relevant model of chronic pain. The second goal was to determine whether there were any sex differences in tolerance to the anti-allodynic effects of ∆^9^-THC in this pain model. We found that disruption of the GRK/β-arrestin-2 pathway of desensitization did not alter sensitivity and/or tolerance to the anti-allodynic effects of either 6 or 10 mg/kg of ∆^9^-THC in a model of cisplatin-evoked neuropathy. We also found that female mice developed tolerance to the anti-allodynic effects of both 6 and 10 mg/kg ∆^9^-THC faster than male littermates.

The finding that the S426A/S430A mutation failed to alter either sensitivity and/or tolerance to ∆^9^-THC was not entirely surprising. Previous studies in our lab have shown that male mice expressing the S426A/S430A mutation show a greater antinociceptive response and/or delayed tolerance development to ∆^9^-THC (Morgan et al., 2014; Henderson-Redmond et al., 2020), CP55,940 (LaFleur et al., 2018; Nealon et al., 2019), and WIN55,212-2 (Nealon et al., 2019) compared to their wild-type littermates in acute models of thermal (tail-flick) and inflammatory (formalin) pain. In contrast, studies examining whether the S426A/S430A mutation can alter tolerance to CB_1_ receptor agonists in a model of cisplatin-evoked neuropathy have been mixed, with the S426A/S430A mutation altering tolerance development to WIN55,212-2 but not CP55,940 (Nealon et al., 2019). However, the delay in tolerance to WIN55,212-2 in the S426A/S430A mutants was much more modest in the chronic pain model than in the acute model (Nealon et al., 2019).

The inability of the S426A/S430A mutation to confer enhanced anti-allodynic effects and/or delayed tolerance to 6 or 10 mg/kg of ∆^9^-THC (a mixed CB_1_/CB_2_ receptor agonist) in these experiments is likely due to the anti-allodynic effects of ∆^9^-THC at CB_2_ receptors which do not undergo tolerance (Deng et al., 2015) and are known to be upregulated in models of chronic pain (Zhang et al., 2003; Wotherspoon et al., 2005; Beltramo et al., 2006). In our previous studies examining the acute antinociceptive effects of ∆^9^-THC, we found that the antinociceptive effects of ∆^9^-THC in the tail-flick assay was exclusively mediated by CB_1_ receptors (Henderson-Redmond et al., 2020). However, mixed CB_1_/CB_2_ receptor agonists have been shown to suppress vincristine-evoked (Rahn et al., 2009) and cisplatin-evoked (Vera et al., 2013) mechanical allodynia through action at both CB_1_ and CB_2_ receptors. Therefore, to better investigate the role of GRK/β-arrestin-2-mediated CB_1_ receptor desensitization in cannabinoid tolerance using the cisplatin model, we used selective CB_1_ and CB_2_ receptor inverse agonists to delineate the contribution of each receptor. We found that pretreatment with the selective CB_1_ receptor inverse agonist rimonabant completely blocked ∆^9^-THC-induced anti-allodynia. However, pretreatment with the selective CB_2_ receptor inverse agonist, SR144528, partially blocks the anti-allodynic effects of ∆^9^-THC, confirming work by other groups showing a greater role of CB_2_ receptors in models of chronic neuropathic vs. acute pain states. Taken together, differential activation of CB_1_ and CB_2_ receptors by different cannabinoid agonists could help explain why we observe differences in the ability of the S426A/S430A mutation to alter anti-allodynic responses to select cannabinoid agonists in mice with cisplatin-evoked neuropathy.

Upregulation of CB_2_ receptors, but not CB_1_ receptors, has been observed in the spinal cord (Zhang et al., 2003; Hsieh et al., 2011) and in dorsal root ganglia (Hsieh et al., 2011) following peripheral nerve injury using chronic constriction injury or spinal nerve ligation approaches. Additional work shows that microglia and astrocyte activation contribute to the onset and maintenance of neuropathic pain (Watkins et al., 2001; Guo et al., 2007) due to their ability to elicit the release of cytokines, including IL-1β, IL-6, and TNF-α, which can enhance pain responses and maintain a neuropathic pain state (Milligan et al., 2001, 2003). Likewise, there is increasing preclinical evidence to suggest that targeting CB_2_ receptors may be more efficacious in alleviating chronic neuropathic pain with fewer side effects than the use of CB_1_ receptor agonists (for a review, see Guindon and Hohmann 2008). Taken together, it is likely that our ∆^9^-THC-induced anti-allodynia is being mediated, at least in part, through CB_2_ receptors.

Peripheral CB_1_ receptors have been shown to modulate neuropathic pain (Fox et al., 2001; Toth et al., 2010; Starowicz et al., 2012; Deng et al., 2015). Mutant mice lacking CB_1_ in peripheral nociceptors revealed that endocannabinoid-induced antinociception was mediated via CB_1_ expressed in these neurons in mouse models of inflammatory and neuropathic pain (Agarwal et al., 2007). Likewise, the endocannabinoid, anandamide, has been shown to reduce cisplatin-induced hyperalgesia through activation of peripheral CB_1_ receptors (Khasabova et al., 2012). Despite evidence of at least partial mediation by CB_2_, pretreatment with the CB_1_ inverse agonist, rimonabant, was able to fully reverse the anti-allodynic effects of 10 mg/kg ∆^9^-THC, highlighting the importance of CB_1_ receptors in a model of cisplatin-evoked neuropathy. Although preclinical work in rodents supports a role for both CB_1_ and CB_2_, through the use of both receptor-selective (Deng et al., 2012; Vera et al., 2013) and/or mixed CB_1_/CB_2_ agonists (Vera et al., 2013; Nealon et al., 2019; Henderson-Redmond et al., 2020) in mediating cisplatin-induced neuropathy, results of clinical work are slightly less clear.

Clinically, studies appear to support a role for cannabinoids, including ∆^9^-THC, for the treatment of chronic, noncancer pain (for a review see, Wong et al., 2020). For example, Nabilone, an FDA approved analog of ∆^9^-THC for the treatment of chemotherapy-induced nausea and vomiting, was found to be superior to both placebo and/or an active control in relieving pain associated with chronic headaches (Pini et al., 2012), diabetic neuropathy (Toth et al., 2012), and Multiple Sclerosis (MS)-induced chronic pain (Turcotte et al., 2015). Likewise, smoked cannabis containing 4% ∆^9^-THC and vaporized cannabis containing either 1.29% or 3.53% ∆^9^-THC were found to be superior to placebo in attenuating MS spasticity and pain (Corey-Bloom et al., 2012) and in managing neuropathic pain in subjects with varying types of neuropathic pain (Wilsey et al., 2013) (for a review see Lynch and Ware, 2015). A second assessment of the efficacy of medical marijuana for noncancer, neuropathic pain found that while medical marijuana provides short-term pain relief on chronic pain, longer studies need to be done to assess whether the analgesic effects of marijuana persist or dissipate with continued use over time (Deshpande et al., 2015).

In contrast, few clinical trials have examined the role of cannabinoids in managing chemotherapy-evoked neuropathy making it difficult to draw conclusions on the efficacy of ∆^9^-THC in managing CENP. For example, a recent study found that use of Sativex®, the oral mucosal spray containing cannabinoids, was not much more effective than placebo alone in patients that had neuropathic pain persisting at least three months post-chemotherapy (Lynch et al., 2014). However, not only was the sample size in this pilot extremely small (16 participants), but five of the participants showed a large (at least a two-point) decrease in pain according to a numerical rating scale for pain intensity (NRS-PI). Further, 10 patients continued in the study extension, and at 3 and 6 months, those patients that continued with the study saw even greater reductions in pain with NRS-PI scores of 6.9 at baseline reduced to 5.0 and 4.2 at 3 and 6 months, respectively (Lynch et al., 2014). The results of this study suggest that clinically, cannabinoids may be especially beneficial to a subset of those suffering from CENP. Interestingly, patients in the previous study were not differentiated based on the type of chemotherapy received. A recent preclinical finding, however, suggests that different cannabinoids (CBD vs. Δ^9^-THC) may be selective in their efficacy for alleviating neuropathies induced by different chemotherapies (King et al., 2017). Taken together, it is possible that different subsets of individuals may be more responsive to different types of cannabinoid-based treatments depending on the type of chemotherapy administered to treat their cancer. One limitation, however, of using animal models to assess the efficacy of ∆^9^-THC and other cannabinoids in treating CENP is that, unlike patients that undergo chemotherapy to treat cancer and subsequently develop neuropathies as a consequence, these animals do not have cancer prior to receiving chemotherapy. Thus, while we can use animal models to gain valuable insight into the potential for cannabinoids (or other drugs) to manage CENP, clinical CENP may be more difficult to treat given the differences in etiology that can occur following the effects of various cancers on the body, ultimately limiting their utility.

Consistent with previous studies, we found that females developed tolerance to the anti-allodynic effects of ∆^9^-THC faster than their male counterparts (Wakley et al., 2014b). Specifically, we found that female wild-type mice were fully tolerant to the effects of 6 mg/kg ∆^9^-THC after 4 days of treatment whereas male mice were not tolerant until day 7 of ∆^9^-THC treatment (Figure 2). Unsure whether this observed effect was confounded due to differences in acute efficacy for the anti-allodynic effects of ∆^9^-THC, we reassessed tolerance using 10 mg/kg ∆^9^-THC, a dose that fully reversed mechanical allodynia in both male and female mice. As with 6 mg/kg ∆^9^-THC, we observed faster tolerance to 10 mg/kg ∆^9^-THC in female mice as they displayed complete tolerance after 4 days while males did not display tolerance until day 6 of 10 mg/kg ∆^9^-THC treatment (Figure 4). Likewise, when seeking to establish differences in tolerance using equally efficacious doses, we confirmed the finding that females developed tolerance to ∆^9^-THC more rapidly than male littermates (Figure 5).

Evidence suggests that sex differences in the antinociceptive effects of Δ^9^-THC might be due to differences in the relative expression of CB_1_ and CB_2_ receptors (Craft et al., 2012). For example, Craft et al. (2012) found that while acute Δ^9^-THC-mediated antinociception was mediated primarily via CB_1_ receptors in male rats, it was mediated by both CB_1_ and CB_2_ receptors in females. Another possibility is that differences in hormonal signaling might modulate establishment of allodynia, cannabinoid response, and tolerance. Preclinical rodent studies showed that testosterone can reduce inflammatory pain (Da Silva et al., 1993), dampen the immune response to experimentally induced inflammatory pain (Gaillard and Spinedi, 1998), and protect against the development of chronic pain development (Fischer et al., 2007; Schertzinger et al., 2018). Ovary intact Sprague-Dawley female rats exhibit elevated tactile allodynia compared to male rats and ovariectoimzed females (Coyle et al., 1995; Coyle et al., 1996) following partial sciatic nerve ligation. Chemotherapy-evoked mechanical allodynia was also enhanced in female Sprague-Dawley rats compared to male rats (Joseph and Levine, 2003). Ovariectomization decreased mechanical allodynia in female rats to a level observed in males while estrogen-replacement therapy restored elevated mechanical allodynia (Joseph and Levine, 2003). These data suggest that testosterone may offer a protective effect while estrogen may predispose females towards developing allodynia following chronic injury.

In contrast, several studies seem to indicate that estrogen, specifically estradiol, may enhance the antinociceptive and anti-allodynic effects of Δ^9^-THC. For example, female rats have been shown to be more sensitive to the antinociceptive effects of Δ^9^-THC compared to males across a host of acute, inflammatory, and chronic pain states (Tseng and Craft, 2001; Craft et al., 2004; Craft and Leitl, 2008; Craft et al., 2012; Craft et al., 2013; Wakley et al., 2015). Likewise, estradiol enhancement of Δ^9^-THC-induced antinociception in mechanical allodynia has been demonstrated in ovariectomized female rats (Craft and Leitl, 2008; Wakley et al., 2014a) and estrus has been shown to enhance the antinociceptive effects of Δ^9^-THC when estrogen levels are higher (Craft and Leitl, 2008; Wakley and Craft, 2011). However, this same group determined that while hormones could alter Δ^9^-THC-mediated antinociception acutely (Craft and Leitl, 2008; Wakley et al., 2014a, Wakley et al., 2015) and that chronic Δ^9^-THC administration could suppress female cycling (Marusich et al., 2015), hormones did not affect tolerance to Δ^9^-THC (Wakley et al., 2015). Thus, the interplay of hormones on pain, cannabinoid-mediated antinociception, and tolerance is a complex issue that future studies should address.

In contrast to our previous studies in acute pain models, disruption of GRK-β-arrestin-2-induced desensitization of CB_1_ receptors failed to alter sensitivity to and/or tolerance to 6 and/or 10 mg/kg ∆^9^-THC in male or female mice within a model of chronic, cisplatin-evoked neuropathic pain. Interestingly, female mice were less sensitive and faster to develop tolerance to the anti-allodynic effects of 6 and 10 mg/kg ∆^9^-THC compared to their male littermates. As such, it is likely that tolerance to cannabinoids is impacted to a much greater extent by CB_2_ receptors in a cisplatin-evoked model of neuropathic pain. Given that women have a higher incidence and often present with a greater prevalence of chronic and neuropathic pain conditions compared to men, we surmise that sex should be thoroughly evaluated when assessing the therapeutic potential of cannabinoids for pain management.

## Data Availability

The raw data supporting the conclusion of this article will be made available by the authors, without undue reservation.

## Abbreviations

ANOVA, analysis of variance; CB, cannabinoid; CB_1_/CB_1_R, type-1 cannabinoid receptor; CB_2_/CB_2_R, type-2 cannabinoid receptor; CBD, cannabidiol; CENP, chemotherapy-evoked neuropathic pain; CUD, cannabinoid use disorder(s); ∆^9^-THC: delta-9-tetrahydrocannabinol; GPCR, G protein-coupled receptor; GRK, G protein-coupled receptor kinase; IP, intraperitoneal; KI, knock-in; MS, multiple sclerosis; NRS-PI, numerical rating scale for pain intensity; SC, subcutaneous; SEM, standard error of the mean; WT, wild-type.
